# Habitual Snoring in school-aged children: environmental and biological predictors

**DOI:** 10.1186/1465-9921-11-144

**Published:** 2010-10-19

**Authors:** Shenghui Li, Xinming Jin, Chonghuai Yan, Shenghu Wu, Fan Jiang, Xiaoming Shen

**Affiliations:** 1 From the Shanghai Xin Hua Hospital affiliated with Shanghai Jiaotong University School of Medicine, Shanghai, People's Republic of China; 2 School of Public Health affiliated with Shanghai Jiaotong University School of Medicine, Shanghai, People's Republic of China; 3 Shanghai Children's Medical Center affiliated with Shanghai Jiaotong University School of Medicine, Shanghai, People's Republic of China; 4 Shanghai Key Laboratory of Children's Environmental Health, Shanghai, People's Republic of China

## Abstract

**Background:**

Habitual snoring, a prominent symptom of sleep-disordered breathing, is an important indicator for a number of health problems in children. Compared to adults, large epidemiological studies on childhood habitual snoring and associated predisposing factors are extremely scarce. The present study aimed to assess the prevalence and associated factors of habitual snoring among Chinese school-aged children.

**Methods:**

A random sample of 20,152 children aged 5.08 to 11.99 years old participated in a cross-sectional survey, which was conducted in eight cities of China. Parent-administrated questionnaires were used to collect information on children's snoring frequency and the possible correlates.

**Results:**

The prevalence of habitual snoring was 12.0% (14.5% for boys vs. 9.5% for girls) in our sampled children. Following factors were associated with an increased risk for habitual snoring: lower family income (adjusted odds ratio [OR] = 1.46), lower father's education (OR = 1.38 and 1.14 for middle school or under and high school of educational level, respectively), breastfeeding duration < 6 months (OR = 1.17), pregnancy maternal smoking (OR = 1.51), obesity (OR = 1.50), overweight (OR = 1.35), several respiratory problems associated with atopy and infection, such as chronic/allergic rhinitis (OR = 1.94), asthma (OR = 1.43), adenotonsillar hypertrophy (OR = 2.17), and chronic otitis media (OR = 1.31), and family history of habitual snoring (OR = 1.70).

**Conclusion:**

The prevalence of habitual snoring in Chinese children was similar to that observed in other countries. The potential predisposing factors covered socioeconomic characteristics, environmental exposures, chronic health problems, and family susceptibility. Compared to socioeconomic status and family susceptibility, environmental exposures and chronic health problems had greater impact, indicating childhood habitual snoring could be partly prevented by health promotion and environmental intervention.

## Introduction

Habitual snoring (HS), a prominent symptom of sleep-disordered breathing (SDB), usually defined as the presence of loud snoring at least three nights per week, is prevalent in children [1-8]. It was reported that the prevalence of HS in school-aged children was ranged from 4.9% to 17.1% in Western countries, such as Italy, Brazil, Germany, Portugal, Australia, and the USA [1-8]. There is a general recognition that HS is an important indicator for a number of health problems in children, including poor physical growth, emotional and behavioral problems, neurocognitive impairment and decreased academic performance, and less often cardiovascular abnormalities [8-13].

While the evidence for the existence of HS faced by many children and an association between HS and its negative consequences is becoming quite impressive, increasing attention should be focused on potential risk factors associated with childhood HS. However, it should be noted that studies, especially large epidemiological studies, on childhood HS and associated risk factors were scare. A few number of studies suggested that the influential factors regarding HS among children were multidimensional, including adenotonsillar hypertrophy, obesity, dental malocclusion, exposure to respiratory infections, cigarette smoking, recurrent otitis media, allergic rhinitis, and lower socioeconomic status [14-18].

It was suggested that HS was physiologically partly determined by craniofacial structures [18]. There was evidence that craniofacial features were marked with racial differences [19]. Therefore, the prevalence and potential predisposing factors regarding HS may vary between different racial groups. Meanwhile, most studies regarding HS focused on children in European-American countries, with much less work being directed at children in Asian countries.

Studies in Thai and Hong Kong districts found that the prevalence of HS in school-aged children was 6.9% and 10.9, respectively [20,21]. Due to a relatively small sample and restricted setting, the data from Thai and Hong Kong districts shouldn't be representative of Chinese children. Therefore, the present epidemiological study was designed to investigate the prevalence of HS and examine the predisposing factors on most of the currently known possible risk factors among a large nationally representative sample of school-aged children in Mainland, China.

## Methods and Materials

### Study design and subjects

Based on a cross-sectional design, 55 elementary schools from eight cities were selected during November and December of 2005, using a cluster-stratified selection procedure. These cities were Urumqi, Chengdu, Xi'an, Hohhot, Wuhan, Canton, Shanghai, and Harbin. For every city, 3-10 districts were randomly selected and within each district, 1-3 elementary schools were chosen. Among these districts and schools, 30 districts and 42 schools were located in urban areas and 9 districts and 13 schools were located in rural/suburban areas. The purposes of this research project were explained to school principals and teachers of the target schools. After the permissions were obtained from these schools, students who were eligible to participate in this study were invited to take the questionnaires on sleep behaviors and personal and family information to their parents, with a cover letter explaining the objectives of the project and instructions on how to complete the questionnaires. Parents were told that the participation was voluntary and informed consent was signed. Of 23,791 children recruited from six grades of the chosen schools, 22,018 (92.5%) returned completed questionnaires.

It was well known that the pubertal development is accompanied by profound changes in biological characteristics, such as craniofacial and larynx structure, which were associated with SDB [22,23]. To eliminate the possible pubertal influences on the results of our study, children who had entered pubertal development were considered to be excluded. To the best of our knowledge, the definition of adolescence was varied between different countries. In China, adolescents usually refer to children aged 12/13 to 17/18 years old [24,25]. Therefore, 1313 children ≥ 12.00 years of age were excluded from the sample. In addition, 536 children were also excluded because of being receiving medication with likely effects on sleep, such as psychostimulants, anticonvulsants, or antihistamines. Finally, 17 (0.1%) children with missing information on frequency of snoring were excluded from further analyses. The final sample consisted of 20,152 children (49.3% boys vs. 50.7% girls). The mean age of the sample was 9.01 years (SD = 1.60 years, range from 5.08 to 11.99 years).

The ethical application of this study was approved by the Ministry of Education of the People's Republic of China.

### Measure

#### Habitual snoring

Sleep behaviors were assessed by a parents-administrated questionnaire -- the Children's Sleep Habits Questionnaire (CSHQ). CSHQ is a 36-item instrument which was designed and developed to assess sleep behaviors of pre-school and school-aged children [26]. In short, the 33 CSHQ items were conceptually grouped into 8 subscales.

A Chinese version of the CSHQ was developed by translation and back translation and has been used previously with proven excellent sensitivity and reliability (Cronbach's alpha's for the internal consistency were 0.73 for the overall questionnaire and ranged from 0.42-0.69 for subscales; Intraclass correlation coefficients for the test-retest reliability were 0.85 for the overall questionnaire and ranged from 0.60-0.88 for subscales; Intraclass correlation coefficients for the parallel reliability were 0.89 for the overall questionnaire and ranged from 0.83-0.92 for subscales) [26].

Subscale of SDB included three items regarding signs and symptoms related to SDB. The internal consistency (Cronbach's alpha) and test-retest reliability (ICCs) of the SDB subscale were 0.68 and 0.76, respectively [27].

Snoring habit was investigated with the question: "How often does your child snore loudly during a typical recent week?" According to the CSHQ, the question was rated on a 3-point scale: "almost always" if occurred 5 to 7 nights per week; "frequently" for 2 to 4 nights per week; and "occasionally/never" for 0 to 1 night per week. For the purpose of this study, children were classified as habitual snorers if the answers were "almost always" or "frequently" and as nonhabitual snorers if the answers were "occasionally/never".

#### Possible risk factors regarding HS

In addition to age and gender, the possible risk factors were conceptually grouped into four domains: socioeconomic status (SES), environmental exposures, chronic health problems, and family member history of SDB.

Socioeconomic variables included parents' educational levels (middle school or under [low], high school [medium], college or above [high]), and household income (< 800, 800-2500, and ≥ 2500 RMB[yuan]/person/month).

Environmental exposure variables included delivery mode (caesarean section/vaginal birth), feeding patterns during the first four months after birth (breastfeeding, mixed feeding, and bottle feeding), duration of breastfeeding (</≥ 6 months), pregnancy maternal smoking (yes/no), and household passive smoking (yes/no).

Children's chronic health problem variables included overweight/obesity status (yes/no, overweight and obesity were defined as body mass index [BMI] [weight in kg/height in m^2^] ≥ 85^th ^and ≥ 95^th ^percentile, respectively), chronic respiratory condition (yes/no, with definition of being ever diagnosed with chronic/allergic rhinitis, asthma, otitis media, or adenotonsillar hypertrophy by pediatricians), and chronic food or drug allergy (yes/no).

Family history of SDB was investigated using the question: "Do the family members (including parents, grandparents, and siblings) habitually snore (yes/no) or were ever diagnosed with OSAS (yes/no)?"

### Statistical Analysis

Statistical descriptions were made by use of the mean, standard deviation for continuous variables, and percentage for categorical variables. Independent-sample *t *test and Chi-square test were used to compare differences between groups where appropriate (Table 1).

**Table 1:** The characteristics for the study sample, Habitual Snorers vs. Nonhabitual Snorers (n = 20,152)

Characteristics N (%)	Total (N = 20152)	Habitual Snorers (N = 2418)	Nonhabitual Snorers (N = 17734)	***t/χ***^***2***^	*P *value
**Sociodemographic characteristics**					
Age (years, mean ± SD)	9.00 ± 1.60	8.88 ± 1.58	9.02 ± 1.61	4.07^a^	<.001
Gender (%)				121.33^b^	
Boys	9890 (49.3)	1437 (59.9)	8445 (47.9)		
Girls	10159 (50.7)	963 (40.1)	9188 (52.1)		
BMI (Kg/m^2^, mean ± SD)	17.33 ± 4.07	17.85 ± 4.40	17.26 ± 4.02	6.18^a^	<.001
Family income (%)				23.99^b^	<.001
< 800	4853 (24.4)	671 (28.2)	4181 (23.9)		
800-2500	11266 (56.6)	1309 (55.0)	9948 (56.8)		
≥ 2500	3793 (19.0)	401 (16.8)	3385 (19.3)		
Mather's education level (%)				1.79^b^	.409
Low	7653 (38.7)	644 (27.4)	4873 (28.0)		
Medium	6616 (33.4)	770 (32.7)	5843 (33.5)		
High	5527 (27.9)	940 (39.9)	6709 (38.5)		
Father's education level (%)				1.20^b^	.548
Low	8433 (42.1)	587 (24.5)	4156 (23.6)		
Medium	6859 (34.2)	802 (33.5)	6050 (34.3)		
High	4750 (23.7)	1008 (42.1)	7422 (42.1)		
**Environmental exposures**					
Delivery				25.24^b^	<.001
Vaginal Birth	13413 (66.8)	1497 (62.3)	11904 (67.4)		
Caesarean section	6658 (33.2)	906 (37.7)	5747 (32.8)		
Feeding patterns during the first four months				11.43	<.001
Breasting feeding	13248 (65.9)	1512 (62.8)	11725 (66.3)		
Mixed/bottle feeding	6869 (34.1)	896 (37.2)	5967 (33.7)		
Breast feeding				13.61^b^	< .001
≥ 6 months	10984 (54.5)	1230 (51.0)	9745 (55.0)		
< 6 months	9168 (45.5)	1182 (49.0)	7978 (45.0)		
Pregnancy maternal smoking	389 (1.9)	76 (3.2)	313 (1.8)	21.42^b^	< .001
Household passive smoking	5000 (24.9)	668 (27.8)	4332 (24.5)	12.43^b^	< .001
**Chronic health problems**					
Obesity	2156 (10.7)	350 (14.6)	1806 (10.2)	37.55^b^	< .001
Overweight	2075 (10.3)	326 (13.5)	1749 (9.8)	24.18^b^	< .001
Chronic or allergic rhinitis	1883 (9.4)	421 (17.5)	1460 (8.2)	212.54^b^	< .001
Asthma	636 (3.2)	138 (5.7)	497 (2.8)	59.08^b^	< .001
Adenotonsillar hypertrophy	2218 (11.0)	498 (20.6)	1716 (9.7)	260.64^b^	< .001
Chronic otitis media	785 (3.9)	141 (5.8)	643 (3.6)	27.93^b^	< .001
Food/drug allergy	1101 (5.5)	174 (7.2)	925 (5.2)	16.35^b^	< .001
**Family history of SDB**					
Habitual snoring	6625 (32.9)	1060 (43.9)	5558 (31.4)	152.43^b^	< .001
OSAS	481 (2.4)	93 (3.9)	388 (2.2)	25.29^b^	< .001

Family income was expressed in RMB(yuan)/person/month

^a ^Independent-samples *t *test

^b ^Chi-square test

To identify risk factors regarding HS in our sampled children, the logistic regression analyses were performed, with "1" for HS and "0" for non-HS. Unadjusted odds ratios (OR) and 95% confidence intervals (CI) for HS were calculated using univariate logistic regression (Table 2). Adjustments were further made by the multivariate regression models following a three-step procedure. Each model included additional variables to assess increasingly proximate determinants of HS. Firstly, a simple model (model I) adjusted only for age and gender (Tables 3 and 4). Secondly, variables regarding socioeconomic characteristics and environmental exposures (Table 3) or health problems and family history (Table 4) were further included (model II). Finally, a full model (model III) was established by adjusting age, gender, all socioeconomic and environmental factors, and all variables regarding health problems and family history simultaneously. The multivariate model included variables retaining significance after a forward likelihood-ratio stepwise elimination procedure. Statistical tests of regression estimates or odds ratio were based on Wald statistics.

**Table 2:** Associated factors regarding habitual snoring by univariate logistical regression models (N = 21,052)

Variables	Prevalence of habitual snoring n (%)	Univariate regression models	
		
		OR (95% CI)	*P *value
**Demographic characteristics**			
Age (years, mean ± SD)			.040
5-6	309 (12.6)	1.24 (1.14-1.47)	.014
7-	491 (13.4)	1.33 (1.14-1.55)	< .001
8-	462 (12.4)	1.22 (1.04-1.42)	.013
9-	433 (11.8)	1.15 (0.98-1.34)	.091
10-	408 (11.2)	1.09 (0.93-1.27)	.309
11-	297 (10.4)	1.00	
Gender (%)			< .001
Boys	1437 (14.5)	1.62 (1.49-1.77)	
Girls	963 (9.5)	1.00	
**Socioeconomic characteristics**			
Family income (%)			< .001
<800	671 (13.8)	1.36 (1.19-1.55)	< .001
800-2500	1309 (11.6)	1.11 (0.99-1.25)	.082
≥ 2500	401 (10.6)	1.00	
Mather's education level (%)			.409
Low	644 (11.7)	0.94 (0.85-1.05)	.284
Medium	770 (11.6)	0.94 (0.85-1.04)	.237
High	940 (12.3)	1.00	
Father's education level (%)			.548
Low	587 (12.4)	1.04 (0.93-1.16)	.354
Medium	802 (11.7)	0.98 (0.88-1.08)	.442
High	1008 (12.0)	1.00	
**Chronic health problems**			
Obesity/overweight			< .001
Obesity	349 (16.2)	1.67 (1.15-1.66)	< .001
Overweight	320 (15.4)	1.57 (1.37-1.81)	< .001
Normal or under	1344 (10.4)	1.00	
Chronic or allergic rhinitis			< .001
Yes	421 (22.4)	2.35 (2.09-2.65)	
No	1990 (10.9)	1.00	
Asthma			< .001
Yes	138 (21.7)	2.10 (1.73-2.55)	
No	2274 (11.7)	1.00	
Adenotonsillar hypertrophy			< .001
Yes	498 (22.5)	2.43 (2.17-2.71)	
No	1914 (10.7)	1.00	
Chronic otitis media			< .001
Yes	141 (18.0)	1.65 (1.37-1.99)	
No	2270 (11.7)	1.00	
Food/drug allergy			< .001
Yes	174 (15.8)	1.41 (1.19-1.67)	
No	2237 (11.8)	1.00	
**Environmental exposures**			
Delivery			< .001
Caesarean sectionh	906 (13.6)	1.25 (1.15-1.37)	
Vaginal Birth	1497 (11.2)	1.00	
Feeding patterns during the first four months			
Breasting feeding	1512 (11.4)	1.00	.001
Mixed/bottle feeding	896 (13.1)	1.16 (1.07-1.27)	
Breast feeding			
≥ 6 months	1230 (11.2)	1.00	< .001
< 6 months	1182 (12.9)	1.21 (1.08-1.56)	
Pregnancy maternal smoking			< .001
Yes	76 (19.5)	1.81 (1.41-2.33)	
No	2333 (11.8)	1.00	
Household passive smoking			< .001
Yes	668 (13.4)	1.19 (1.08-1.31)	
No	1735 (11.5)	1.00	
**Family history of SDB**			
Habitual snoring			< .001
Yes	1060 (16.0)	1.72 (1.57-1.87)	
No	1352 (10.0)	1.00	
OSAS			< .001
Yes	93 (19.3)	1.79 (1.42-2.26)	
No	2319 (11.8)	1.00	

Family income was expressed in RMB(yuan)/person/month

OR: odds ratio; CI: confidence interval

**Table 3:** Socioeconomic and environmental factors regarding habitual snoring by multivariate logistical regression models (N = 21,052)

Variables	Model I		Model II		Model III	
			
	Adjusted OR (95% CI)	*P *value	Adjusted OR (95% CI)	*P *value	Adjusted OR (95% CI)	*P *value
**Socioeconomic characteristics**						
Family income		< .001		< .001		< .001
< 800 vs. ≥ 2500	1.35 (1.18-1.54)	< .001	1.43 (1.23-1.67)	< .001	1.46 (1.23-1.75)	< .001
800-2500 vs. ≥ 2500	1.12 (0.99-1.26)	.072	1.16 (1.02-1.32)	.024	1.12 (0.96-1.30)	.142
Mather's education level						
Low vs. High	NS		NS		NS	
Medium vs. High	NS		NS		NS	
Father's education level				.007		< .001
Low vs. High	NS		1.22 (1.08-1.38)	.002	1.38 (1.20-1.60)	< .001
Medium vs. High	NS		1.04 (0.94-1.16)	.427	1.14 (1.01-1.29)	.034
**Environmental exposures**						
Delivery						
Caesarean section vs. Vaginal Birth	1.21 (1.11-1.33)	< .001	1.19 (1.08-1.31)	< .001	NS	
Feeding patterns during the first four months						
Mixed/bottle feeding vs. Breastfeeding	1.16 (1.06-1.27)	.001	NS			
Breastfeeding						
<6 months vs. ≥ 6 months	1.19 (1.09-1.29)	< .001	1.14 (1.05-1.25)	.003	1.17 (1.08-1.28)	< .001
Pregnancy maternal smoking						
Yes vs. No	1.81 (1.42-2.38)	< .001	1.68 (1.28-2.21)	< .001	1.51 (1.07-2.13)	.019
Household passive smoking						
Yes vs. No	1.18 (1.07-1.30)	.001	1.16 (1.05-1.29)	.003	NS	

Family income was expressed in RMB(yuan)/person/month

OR: odds ratio; CI: confidence interval

Model I adjusted for age and gender;

Model II adjusted for age, gender, and all socioeconomic and environmental factors

Model III adjusted for age, gender, all socioeconomic and environmental factors, and all health problems and family history simultaneously.

**Table 4:** Chronic health problems and family history regarding habitual snoring by multivariate logistical regression models (N = 21,052)

Variables	Model I		Model II		Model III	
			
	Adjusted OR (95% CI)	*P *value	Adjusted OR (95% CI)	*P *value	Adjusted OR (95% CI)	*P *value
**Demographic characteristics**						
Age (years, mean ± SD)		.008		< .001		< .001
5-6 vs. 11-	1.23 (1.04-1.46)	.015	1.50 (1.23-1.84)	< .001	1.53 (1.25-1.88)	< .001
7- vs. 11-	1.32 (1.13-1.54)	< .001	1.44 (1.20-1.73)	< .001	1.47 (1.21-1.77)	< .001
8- vs. 11-	1.19 (1.02-1.39)	.027	1.28 (1.06-1.53)	.010	1.29 (1.07-1.56)	.008
9- vs. 11-	1.15 (0.98-1.34)	.091	1.16 (0.96-1.40)	.134	1.18 (0.97-1.44)	.090
10- vs. 11-	1.08 (0.92-1.27)	.328	1.15 (0.95-1.39)	.167	1.15 (0.95-1.40)	.163
Gender						
Boys vs. Girl	1.62 (1.48-1.76)	< .001	1.53 (1.38-1.69)	< .001	1.55 (1.40-1.72)	< .001
**Chronic health problems**						
Obesity vs. normal or under	1.54 (1.35-1.78)	< .001	1.51 (1.33-1.76)	< .001	1.50 (1.31-1.74)	< .001
Overweight vs. normal or under	1.44 (1.25-1.66)	< .001	1.38 (1.20-1.60)	< .001	1.35 (1.16-1.56)	< .001
Chronic/allergic rhinitis vs. none	2.27 (2.01-2.56)	< .001	1.97 (1.70-20.27)	< .001	1.94 (1.66-2.25)	< .001
Asthma vs. none	1.99 (1.64-2.42)	< .001	1.46 (1.14-1.87)	.002	1.43 (1.11-1.84)	.006
Adenotonsillar hypertrophy vs. none	2.35 (2.10-2.63)	< .001	2.12 (1.86-2.43)	< .001	2.17 (1.90-2.49)	< .001
Chronic otitis media vs. none	1.60 (1.32-1.93)	< .001	1.31 (1.05-1.64)	.017	1.31 (1.06-1.65)	.021
Food/drug allergy vs. none	1.38 (1.16-1.64)	< .001	NS		NS	
**Family history of SDB**						
HS vs. none	1.75 (1.60-1.91)	< .001	1.67 (1.51-1.85)	< .001	1.70 (1.52-1.89)	< .001
OSAS vs. none	1.81 (1.43-2.28)	< .001	NS		NS	

OR: odds ratio; CI: confidence interval

Model I adjusted for age and gender;

Model II adjusted for age, gender, and all health problems and family history

Model III adjusted for age, gender, all socioeconomic and environmental factors, and all health problems and family history simultaneously.

All analyses were performed using the Statistical Package for Social Sciences (SPSS) for Windows, version 12.5 (SPSS Inc, Chicago, IL, USA). In the presentation of the results, the statistical significance was set at P value < .05 (two tailed).

## Results

### Prevalence of HS and characteristics of the sample

**Figure 1:**
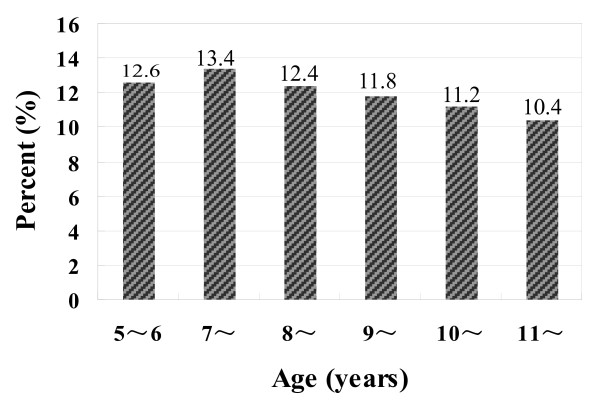
**The prevalence of HS in Chinese school-aged children (n = 20,152)**.

Our survey showed that the prevalence of HS in our sampled children was 12.0%. Significantly gender difference was found with boys higher prevalent (14.5% vs. 9.5%; *χ*^*2 *^= 121.33, p < .001). An interesting age differences were also found: first significantly increased from 5-6 to 7 years and then gradually declined (*χ*^*2 *^= 18.09, p = .004). Figure 1 showed the prevalence of HS by age.

Table 1 summarized the sample characteristics stratified by habitual snorers vs. nonhabitual snorers. Compared with nonhabitual snorers, habitual snorers were significantly younger, had higher BMI, and lower family income (all p < .001). In addition, all chronic health problems, caesarean section, mixed/bottle feeding during the first four months after birth, breastfeeding < 6 months, cigarette smoking exposure, and family history of SDB were more common in habitual snorers (all p < .001).

### Predisposing factors of HS by logistical analyses

The unadjusted OR with 95% CI of possible risk factors for HS were demonstrated in Table 2. It can be seen that, except for parental educational levels, all other factors were significantly associated with HS in the univariate regression models.

#### Socioeconomic characteristics and environmental exposures

The association between HS and socioeconomic and environmental factors was shown in Table 3. After adjusting only for age and gender, those factors, such as lower family income, caesarean section, mixed/bottle feeding during the first four months after birth, breastfeeding < 6 months, pregnancy maternal smoking, and household passive smoking were significantly associated with an increased likelihood of HS (Model I). After adjusting for socioeconomic factors and environmental exposures simultaneously, these six factors remained statistically significant (Model II). Moreover, father's educational level, which was not a significant predictor in Model I, was found to be related to HS in Model II. After adjusting further for all health problem and family history, four factors remained to be independent predictors of HS: lower family income, lower father's educational level, breastfeeding < 6 months, and pregnancy maternal smoking (Model III).

The association between pregnancy maternal smoking and HS was stronger in girls than in boys (OR = 2.46 for girls, OR = 1.16 for boys; p for interaction = .005).

#### Health problems and family history

The association between HS and health problems and family history was shown in Table 4. After adjusting only for age and gender, all eight factors (overweight, obesity, chronic/allergic rhinitis, asthma, otitis media, adenotonsillar hypertrophy, food/drug allergy, family history of HS and OSAS) were significantly associated with an increased likelihood of HS (Model I). After controlling simultaneously for health problems and family history, except for chronic food/drug allergy and family history of OSAS, all other six factors remained statistically significant (Model II). These associations were not found to be changed after further adjusting for socioeconomic factors and environmental exposures, indicating these six factors were independent risk factors for HS in our sampled children (Model III).

The strength of association between asthma and HS varied between different age groups (OR = 2.42 for 5-6 years, OR = 1.94 for 7 years; OR = 1.45 for 8 years; OR = 1.28 for 9 years; OR not remained significant for 10 and 11 years; p for interaction = .003).

## Discussion

Based on a large nationally representative sample, this study demonstrated that the prevalence of HS, defined as loud snoring at least two nights per week, was 12.0% in our sampled Chinese school-aged children. The factors associated with HS covered several domains: socioeconomic characteristics, environmental exposures, chronic health problems, and family history.

### Prevalence of HS in school-aged children

To the best of our knowledge, this was the largest epidemiological study on childhood HS (n = 20,152). Due to a large sample recruited from eight cities with geographical and socioeconomic diversity and a good response rate (92.5%), the results of this study entailed extended information for understanding childhood HS and the correlates.

The prevalence of HS in our sample was 12.0%, which was slightly higher than that reported in Thai (sample aged 6 to 13 years) and Hong Kong (sample aged 6 to 12 years) districts (6.9% and 10.9, respectively) [20,21]. There was evidence that SDB was higher prevalent in younger children than in the older because of the higher volumetric adenoids/rhinopharynx ratio, with a peak between 2 and 8 years [28]. Compared to studies in Thai and Hong Kong districts, our studied sample was slightly younger (aged 5.08 to 12 years). In addition, the definition criteria of HS in our study was a little mild than that the two studies adopted. Taken together, the different age groups and definition of HS may explain, at least partly, the discrepancy in the prevalence of HS.

In consistent to the study in Hong Kong, our study demonstrated that boys were liable to have HS [21]. However, the gender difference was not found in the study of Thai district [20]. An interesting age differences in the prevalence of HS were found: first significantly increased from 5-6 to 7 years old and then gradually declined (12.6%-13.4%-10.4%). The specific waving prevalence of HS from 5-6 to 11 years has not previously been reported and may be explained, or at least partly, by the age-dependent biological development in size of adenoid and changes in atopic diseases during childhood [29,30]. More studies are needed to assess the age difference, which may be valuable in exploring the biological predisposing factors regarding childhood HS.

### Association with socioeconomic characteristics and environmental exposures

The present study revealed that lower fathers' educational level and lower family income were independent predisposing factors for HS. The association between SES and HS has been previously reported and the results were very similar to the findings of our study [17,20,31,32]. In addition, a recent study also found that single parent and overcrowded household could increase the risk of childhood HS [17]. Taken together, disadvantaged SES was an important predictor for childhood HS.

Previous studies showed that there was an association between childhood snoring and smoking exposure [17,20,30,32]. Moreover, a study in preschool children confirmed a dose-dependent effect of household smoking exposure on HS [17]. Our study demonstrated that both pregnancy maternal smoking and household passive smoking were associated with HS after adjusting for socioeconomic characteristics and environmental exposures. However, only pregnancy maternal pregnancy remained significant after further adjusting for health problems and family history and the association was stronger in girls than in boys. In fact, children with prenatal smoking exposure may currently expose to household smoking. Therefore, we could not simply exclude the effect of household smoking on childhood HS. Our results suggested that girls were more vulnerable to smoking exposure, which was a new finding and worth further research.

It was an interesting finding that breastfeeding duration was associated childhood HS. In consistent to the results of our study, a study in preschool children similarly found that longer duration of breastfeeding was a protective factor to HS, although the association did not remained significant after controlling for parental smoking [17]. However, contrary to the result of our study, a study in Singapore children aged 4-7 years suggested that breastfeeding was a risk factor to HS [30]. The contradictory results should be further interpreted or confirmed by longitudinal studies.

### Association with chronic health problems and family history

In our study, a strong association was found between HS and several respiratory problems associated with atopy and infection, including chronic/allergic rhinitis, asthma, adenotonsillar hypertrophy, and otitis media, which has been previously reported [14,15,17,18,30].

The mechanism underline respiratory problems and HS has not been clearly interpreted yet. A number of studies tried to explore and clarify the mechanism [29-32]. In brief, respiratory problems could increase upper airway resistance and affect airway compliance and consequently resulted to HS [33,34]. In turn, HS may exacerbate some respiratory problems such as asthma by increasing cholinergic tone and promote bronch constriction [35]. In addition, a recent study showed early exposure to respiratory syncytial virus might induce neuro-immunomodulatory changes within adenotonsillar tissue [36]. In one word, respiratory problems and HS may be linked through some unknown intrinsic mechanisms, in which airway inflammation was irritated and neuromuscular control of breathing was disturbed.

Accumulating studies indicated that obesity was an independent predisposing factor for childhood HS [32,33,37], which was in accordance to the results of this study. Moreover, our study found a dose-response relationship in that the OR values became greater as the weight status increased (OR = 1.50 for obesity and OR = 1.37 for overweigh). In addition, there was evidence that the relationship between HS and obesity varied between different age groups and the strength was stronger in older children [32,38]. However, our study did not find this age-dependent change (p for interaction >.05).

Our study also found that family history of HS was another strong risk factor for childhood HS. Therefore, it was hypothesized that HS might result from an interaction between underlying host predisposition, various intrinsic mechanisms, and external triggers. That was to say, HS was the combined outcome of environment and heredity.

### Limitations

The present study was limited by the reliance on a subjective measure, which may increase the possibility of rater biases. Fortunately, previous study has shown a high agreement between parental reports and polysomnography recording of snoring frequency [39]. In addition, although polysomnography recording was the standard method for recording of snoring frequency, it maybe not appropriate for such a large population survey. Secondly, compared to habitual snoring, researches on obstructive sleep apnea (OSA) maybe have stronger clinical significance. However, a more recent study indicated that, as the predictive symptoms for OSA, childhood HS, even without apnea, must now be paid close attention and children with HS must be considered to be an at-risk population [40]. Therefore, the present study retained updating clinical significance. Thirdly, although our study included a large number of possible risk factors of HS, the analysis may have been imperfect and non-comprehensive. For example, a more recent study reported that traffic exposure was a risk factor to childhood HS [17]. Due to the fact that we did to collect information on traffic exposure during the survey, it was impossible to assess the relationship between traffic exposure and HS in our sampled children. Moreover, some unknown factors related to HS may responsible for part of the associations reported herein. Finally, since there was evidence that SDB was higher prevalent in younger children than in the older because of the higher volumetric adenoids/rhinopharynx ratio [28], the findings of our study could not be extended to younger children.

## Conclusions

This study provided information on the prevalence of HS and associated risk factors in Chinese school-aged children. Our findings suggested that HS was common in school-aged children and associated factors covered socioeconomic status, environmental factors, and biological susceptibility. Upon the recognition that HS has potential severe complications due to increased sleep fragmentation, theses findings, although should be further confirmed by prospective studies, had important clinical implication for formulating intervention and treatment schemes.

## Abbreviations

HS: habitual snoring; SDB: sleep-disordered breathing; CSHQ: the Children's Sleep Habits Questionnaire; SES: socioeconomic status; BMI: body mass index; SE: standard error; OR: odds ratio; CI: confidence interval.

## Disclosure Statement

All authors indicate no potential conflicts of interests.

## Authors' contributions

SL participated in the design, analysis, interpretation and drafted the manuscript. XS participated in the design and coordination of the study, acquisition of data and to critically draft the manuscript. XJ, CY, SW and FJ participated in the design, acquisition of data and to critically draft the manuscript. All authors read and approved the final manuscript.
